# The intervertebral discs’ fibrocartilage as a DNA source for genetic identification in severely charred cadavers

**DOI:** 10.1007/s12024-022-00536-8

**Published:** 2022-10-08

**Authors:** Marcin Tomsia, Kornelia Droździok, Przemysław Banaszek, Michał Szczepański, Artur Pałasz, Elżbieta Chełmecka

**Affiliations:** 1 Department of Forensic Medicine and Forensic Toxicology, Medical University of Silesia, Medyków 18, 40-752 Katowice, Poland; 2 Department of Histology, Faculty of Medical Sciences, Medical School of Silesia in Katowice, Medyków 18, 40-752 Katowice, Poland; 3 Department of Statistics, Department of Instrumental Analysis, Faculty of Pharmaceutical Sciences, Medical University of Silesia, Ostrogórska 30, 41-200 Sosnowiec, Poland

**Keywords:** Charred cadavers, DNA degradation index, Fibrous cartilage, Fibrous ring, Genetic identification, Intervertebral disc

## Abstract

Identifying charred human remains poses a challenge to forensic laboratories. High temperature completely incinerates the superficial tissues and partially destroys bones, forcing the forensics to seek an alternative, for bones and teeth, forensic material that should quickly and cheaply deliver DNA of sufficient quantity and quality. We sought, other than rib cartilage, types of cartilages that could serve as a DNA source. DNA was isolated from the fibrous cartilage of a fibrous ring of intervertebral L1-L2 discs sampled from charred cadavers or charred body fragments: 5 victims of car fires, 1 victim of combustion during a residential house gas explosion, and 3 victims of nitroglycerin explosion. DNA was isolated by the column method. DNA quality and concentration were assessed by RT-PCR and multiplex PCR for 23 autosomal and 17 Y chromosome STR loci. STR polymorphism results obtained by capillary electrophoresis served for likelihood ratio (LR) calculations. DNA concentration in relation to the cadaver’s age and post-mortem interval (PMI) were analyzed. All samples (*n* = 9) yielded good-quality DNA in quantities (0.57–17.51 ng/µL for T. Large autosomal sequence) suitable for STR-based amplification. The isolated DNA characterized a low degradation index (0.80–1.99), and we were able to obtain complete genetic profiles. In each of the nine cases, the genotyping results allowed identifying the victims based on comparative material from the immediate family. The results demonstrate the usefulness of human intervertebral disc fibrocartilage as an alternative DNA source for the genetic identification of charred bodies or charred torso fragments.

## Introduction

The research on cartilage tissue applications and use in forensic science continues to grow. So far, it has been used for age prediction based on differences in the pigmentation of its cross-section [1], CT images [2], and using the aspartic acid racemization method [3, 4]. Additionally, it has been used for ethanol [5] and other volatile substances [6] or sodium nitrite [7] detection. Cartilage tissue can also be used for DNA profiling [8, 9], even in highly charred cadavers [10]. Although these works were mainly carried out on the vitreous cartilage, which occurs in costal cartilage, the fibrous cartilage, which is part of the intervertebral discs, has also attracted the interest of forensic geneticists [11]. Forensic specialists are challenged to work on human remains subjected to various environmental factors, including extremely high temperatures. The challenge is a driving force to search for optimal biological materials for post-mortem genetic analyses [12]. Apart from searching for “the best tissue,” they also attempt to find the optimal location for collecting material during an autopsy [12] and optimal isolation methods for burnt biological material [13]. In the case of burnt corpses, hard tissue is often the only material available for examination. In such cases, the successful DNA isolation strictly depends on the stage of fire-induced destruction: well preserved, semi-burnt, black burnt, blue-grey burnt, blue-grey-white burnt) [14]. Becker et al. [11] have shown that the intervertebral discs are an excellent source of DNA, resistant to various environmental factors, and have been able to use it in a case where a fire caused death. However, the use of fibrous cartilage of the intervertebral discs from charred cadavers or charred parts of the human body for DNA profiling has not yet been studied. In such situations, DNA isolation from bone tissues is also possible, but using bone tissue can be more time-consuming, labor-intensive, or less cost-effective depending on the method.

The presented paper aims to emphasize the possibility of using the fibrous cartilage of the intervertebral discs as a source of DNA in individual identification in situations where the corpse or body fragments are charred.

## Material and methods

### Ethics approval

The study was approved by the Bioethical Commission of Medical University of Silesia in Katowice (decision no. PCN/CBN/0052/KB/77/22; approval date: 5 May 2022).

### Sample collection

The intervertebral discs were collected during 9 medico-legal autopsies. The post-mortem interval ranged between 2 and 10 days (7.0 ± 2.9 days). Six samples were collected from charred bodies (males) and three from charred spines’ fragments (Table 1). All tissue samples were stored at − 20 °C until further processing.

**Table 1:** Death circumstances, age, gender of the cadavers and DNA concentrations, and DNA degradation index of the intervertebral discs sampled from nine charred cadavers (cases 1–6) or charred body fragments (cases 7–9). The results are presented as mean ± standard deviation

**Case no.**	**1**	**2**	**3**	**4**	**5**	**6**	**7**	**8**	**9**
**Death circumstances**	Self-immolation in the car	Self-immolation in the car	Car fire	Car fire	Self-immolation in the car	Construction disaster after gas explosion	Uncontrolled detonation of nitroglycerin	Uncontrolled detonation of nitroglycerin	Uncontrolled detonation of nitroglycerin
**T of the flame (°C)**	1100	1000	< 660	N/A	N/A	N/A	N/A	N/A	N/A
**Gender**	Male	Male	Male	Male	Male	Male	Male*	Male*	Male*
**Age of the cadaver (years)**	45	26	55	47	63	8	43	43	43
**PMI (days)**	6	9	7	5	2	6	10	10	10
**T. Large autosomal DNA concentration (ng/µL)**	4.11 ± 0.31	5.60 ± 0.45	9.38 ± 0.31	3.03 ± 0.09	0.57 ± 0.04	17.51 ± 1.50	5.34 ± 0.31	4.94 ± 0.45	10.07 ± 0.40
**T. Small autosomal DNA concentration (ng/µL)**	3.65 ± 0.58	4.48 ± 0.53	9.06 ± 0.39	2.79 ± 0.63	0.72 ± 0.14	34.77 ± 2.63	6.17 ± 0.29	4.18 ± 0.47	9.02 ± 0.71
**T. Y DNA concentration (ng/µL)**	5.00 ± 0.22	5.95 ± 0.19	10.86 ± 0.65	4.17 ± 0.31	0.94 ± 0.15	25.02 ± 2.05	8.05 ± 0.41	5.73 ± 0.56	12.32 ± 0.74
**DNA degradation index**	0.88 ± 0.08	0.80 ± 0.05	0.97 ± 0.03	0.92 ± 0.18	1.26 ± 0.20	1.99 ± 0.07	1.16 ± 0.11	0.85 ± 0.05	0.90 ± 0.11

*N/A* not available, *PMI* post-mortem interval

*Cases with only spine fragment available for analyses

**Fig. 1:**
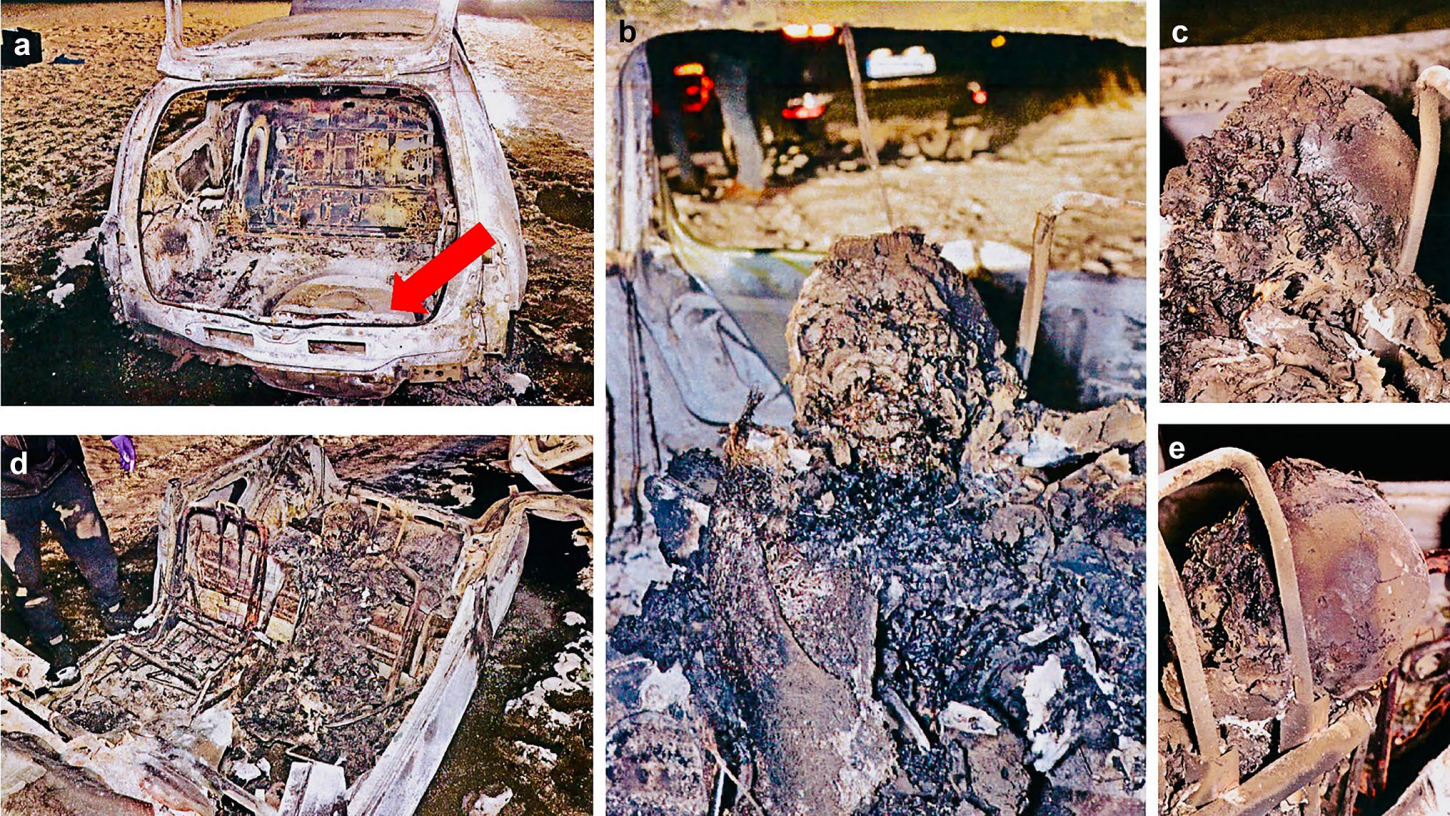
The charred corpse reveal site (case 1): **a** the interior of the trunk compartment, after lifting the trunk door and a fragment of the trunk floor — the red arrow points to the gas tank, **b** charred skull with the body front, **c** and **e** charred skull on both sides, **d** the interior of the vehicle with the visible corpse after removing the roof

**Fig. 2:**
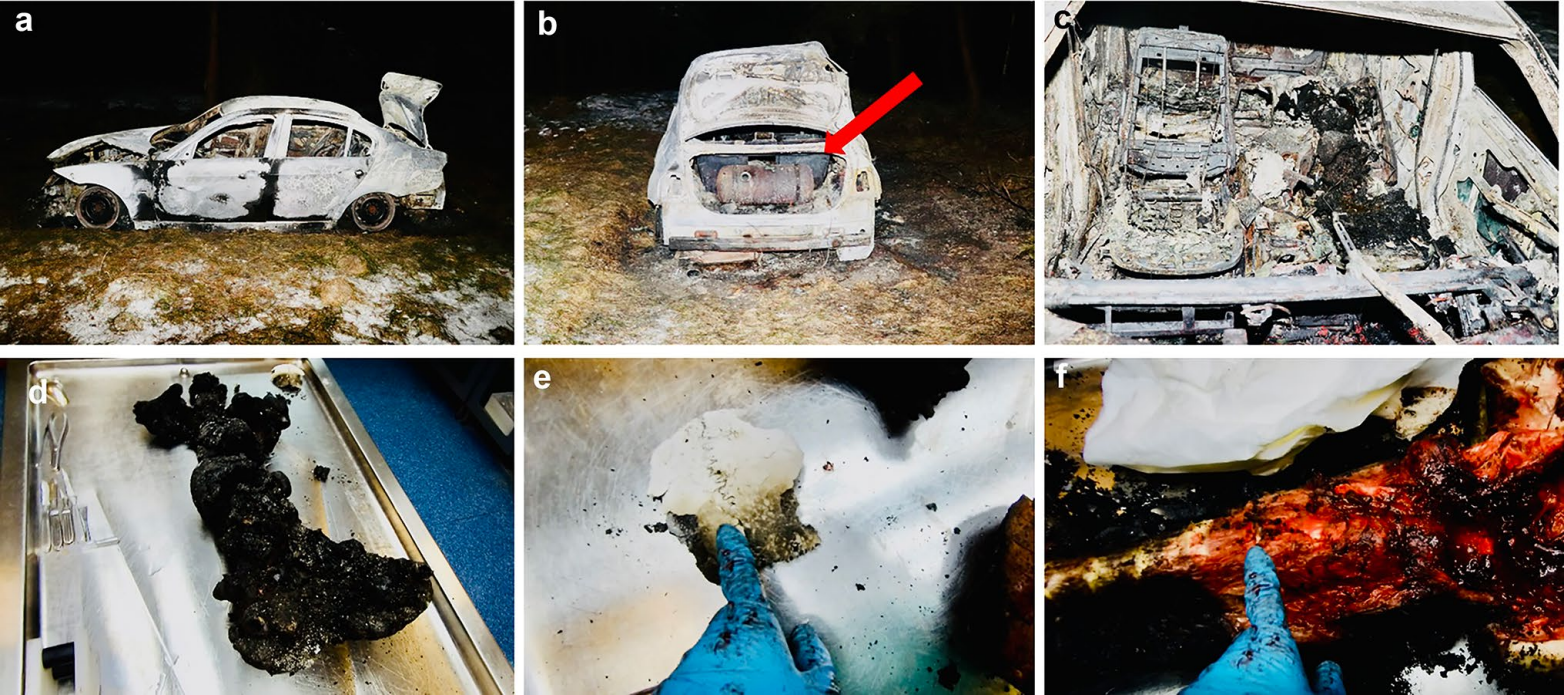
The charred corpse reveal site and internal examination (case 2): **a** side view of the car wreckage; **b** rear view of the burnt vehicle — the red arrow marks the LPG fuel tank, **c** the interior of the vehicle with charred human remains, **d** charred human remains, **e** fragment of the burnt skull vault revealed at the incident site and delivered to the dissecting room together with the corpse, **f** visual inspection of the charred corpse’s spine during the forensic medical autopsy — the technician indicates the location (L1-L2) of the fibrous ring sampling for genetic testing

**Fig. 3:**
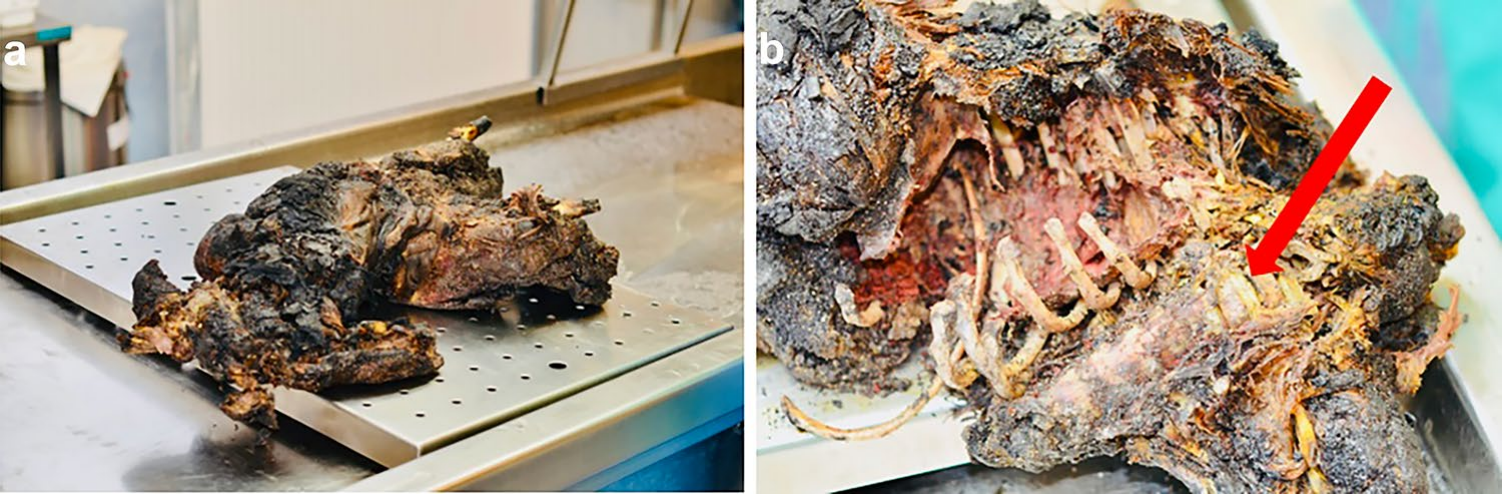
The corpse revealed at the fire site and a construction disaster after a gas explosion in a single-family house (case 6): **a** charred body, **b** the location of the intervertebral disc sampling

**Fig. 4:**
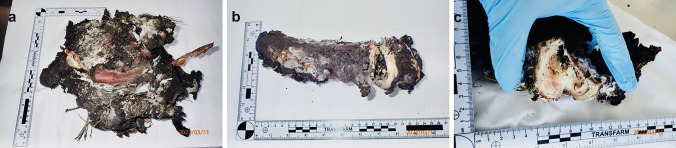
Three charred tissue fragments with the spine parts revealed after an uncontrolled nitroglycerin explosion: **a** case 7, **b** case 8, and **c** case 9

In five cases, the charred body was revealed in the car wreckage (Figs. 1 and 2). In one case, the charred body was revealed in the rubble of a burnt down and collapsed house after a gas explosion (Fig. 3). The three spine fragments were revealed during the site examination after an uncontrolled nitroglycerin explosion (Fig. 4). The victims’ age ranged from 8 to 63 years (43.4 ± 16.0 years).

### Hematoxylin–eosin histochemical staining

**Fig. 5:**
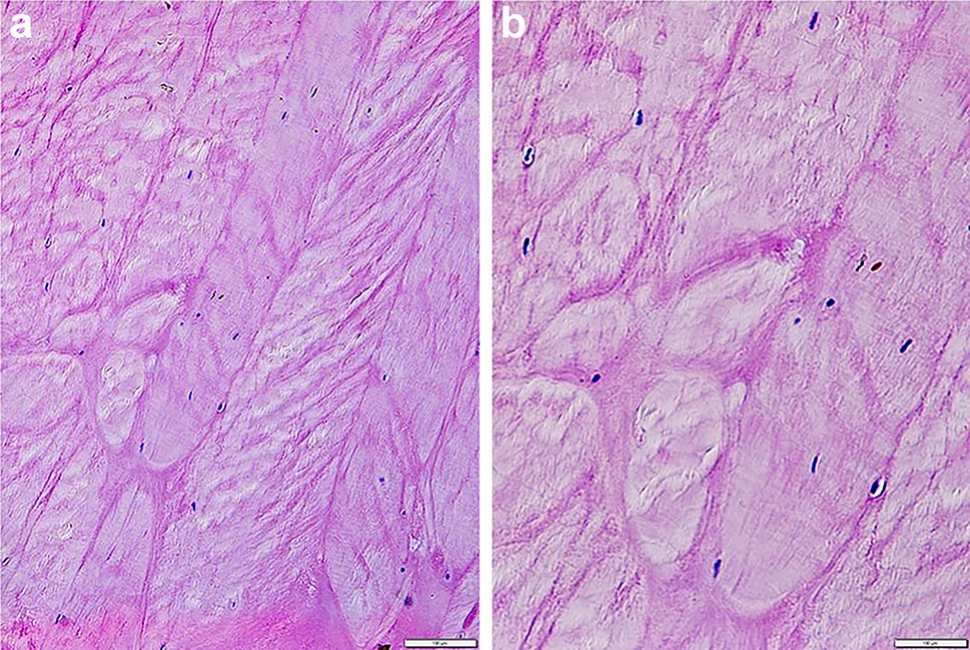
Fibrous cartilage of a fibrous ring of an intervertebral disc taken from a charred corpse (case 3): **a** magnification 10 × , **b** magnification 20 × . The scale is 100 µm. Purple parts are cell nuclei stained with hematoxylin–eosin

Annulus fibrosus fragments were collected during forensic autopsy from the cadaver (case 1) or from charred spine fragment (cases 7–9). The discs were cut with a sterile surgical scalpel from the L1-L2 lumbar region due to the greater thickness and ease of preparation compared to the spine upper sections. The external surface of the collected fragment and contaminations were cleaned also with a sterile surgical scalpel. Tissue fragments were fixed in 4% formalin for 48 h and then fixed in a paraffin block. The tissue was sliced into 3-µm-thick samples on a microtome. The hematoxylin–eosin (H&E) histological staining [15, 16] was performed to visualize the cell nuclei (Fig. 5) in the examined tissue in order to confirm that the genetic test is well-founded.

### DNA extraction

**Fig. 6:**
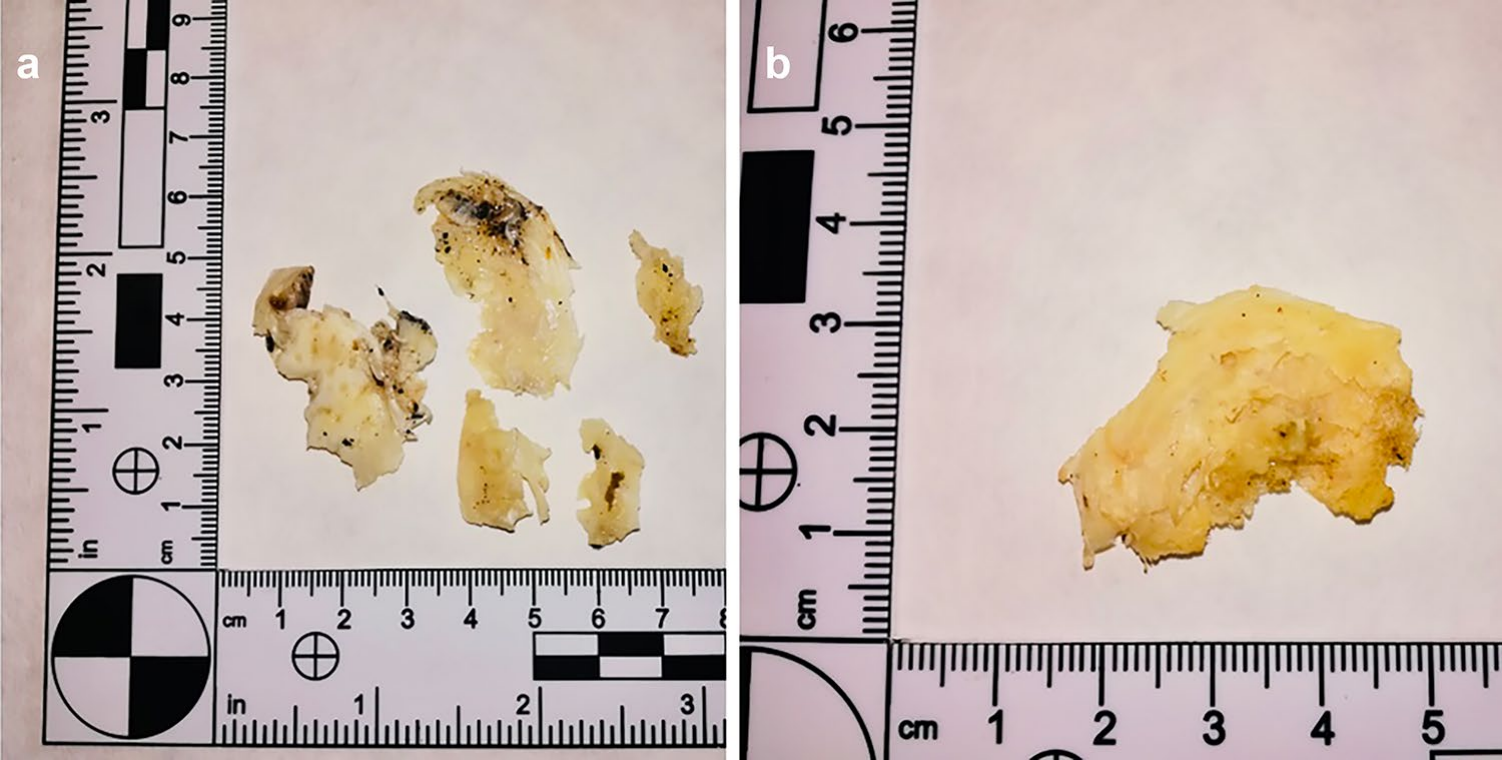
Fragments of a fibrous ring collected from a charred corpse (case 3): **a** fragments with visible soot traces, **b** the other side of one of the fragments presented in photo **a**

Clean annulus fibrosus fragments (Fig. 6) were cut into 3 × 2 mm pieces with a sterile scalpel blade (Fig. 1b). DNA isolation was performed using a Sherlock AX kit (A&A Biotechnology, Poland) according to the manufacturer’s instruction. The final volume of the DNA solution used was 50 µL.

### Real-time PCR

The quality and concentration of DNA samples were evaluated in triplicate using a Quantifiler™ Trio DNA Quantification kit (Applied Biosystems, USA), HiD v. 1.2 software, and 7500 Real-Time PCR System (Applied Biosystems, USA). The qualitative analysis was performed using sequences for T. Large (214 bp) and T. Small (80 bp) autosomal chromosomes and the Y chromosome (75 bp). The degradation index was calculated based on the ratio of T. Small to T. Large autosomal sequences. All procedures were executed according to the manufacturer’s recommendations.

### Multiplex PCR and capillary electrophoresis

PCR was performed using a PowerPlex ESX 17 and PowerPlex HS 16 kit (Promega Corporation, USA) in a Gene Amp PCR System 9700 thermocycler (Applied Biosystems, USA). Amplification products were separated towards DNA CC5 ILS 500 and CC5 ILS 600 standards (Promega Corporation, USA) using a 3130 Genetic Analyzer (Applied Biosystems, USA). The following loci were analyzed: AMEL, D3S1358, TH01, D21S11, D18S51, D10S1248, D1S1656, D2S1338, D16S539, D22S1045, VWA, D8SS1179, FGA, D2S441, D12S391, D19S433, SE33, D5S818, D13S317, D7S820, TPOX, CSF1PO, Penta D, and Penta E. Additionally, alleles from chromosome Y were determined using a Yfiler test (Applied Biosystems, USA). PCR products were analyzed in a 3130 Genetic Analyzer (Applied Biosystems, USA). Genotypes were generated using Gene Mapper ID v3.2 software (Applied Biosystems, USA). Multiplex PCR and capillary electrophoresis procedures were executed according to the manufacturer’s recommendations.

### Statistical analysis

The likelihood ratio (LR) was calculated using DNA Stat software v. 2.1 (Laser Systemy Informatyczne S.A., Poland) [17]. The remaining analyses were done using Statistica, version 13 (TIBCO Software Inc., Palo Alto, USA). Data distribution was tested using the Shapiro–Wilk test and quantile–quantile plot analysis. Data with normal distribution were presented as mean ± standard deviation. Linear regression analysis was used to assess the relationship between the analyzed parameters.

## Results

Histological analysis of the H&E-stained samples (Fig. 3, case 3) confirmed that the collected tissue was fibrocartilage. The analysis also showed the presence of cell nuclei and, consequently, the validity of qualifying the sample for genetic testing.

### Quantification and DNA profile quality analysis

The quantity and quality of the isolated DNA qualified all samples for genotyping. However, the quantity was very variable between samples: from 0.57 to 17.51 ng/µL for T. Large autosomal sequence, from 0.72 to 34.77 ng/µL for T. Small autosomal sequence, and from 0.94 to 25.02 ng/µL for the Y chromosome sequence. The degradation index ranged between 0.80 and 1.99, and in all cases it fit within the kit’s specific threshold. The detailed information about the quantity and quality of isolated DNA is summarized in Table 1.

**Fig. 7:**
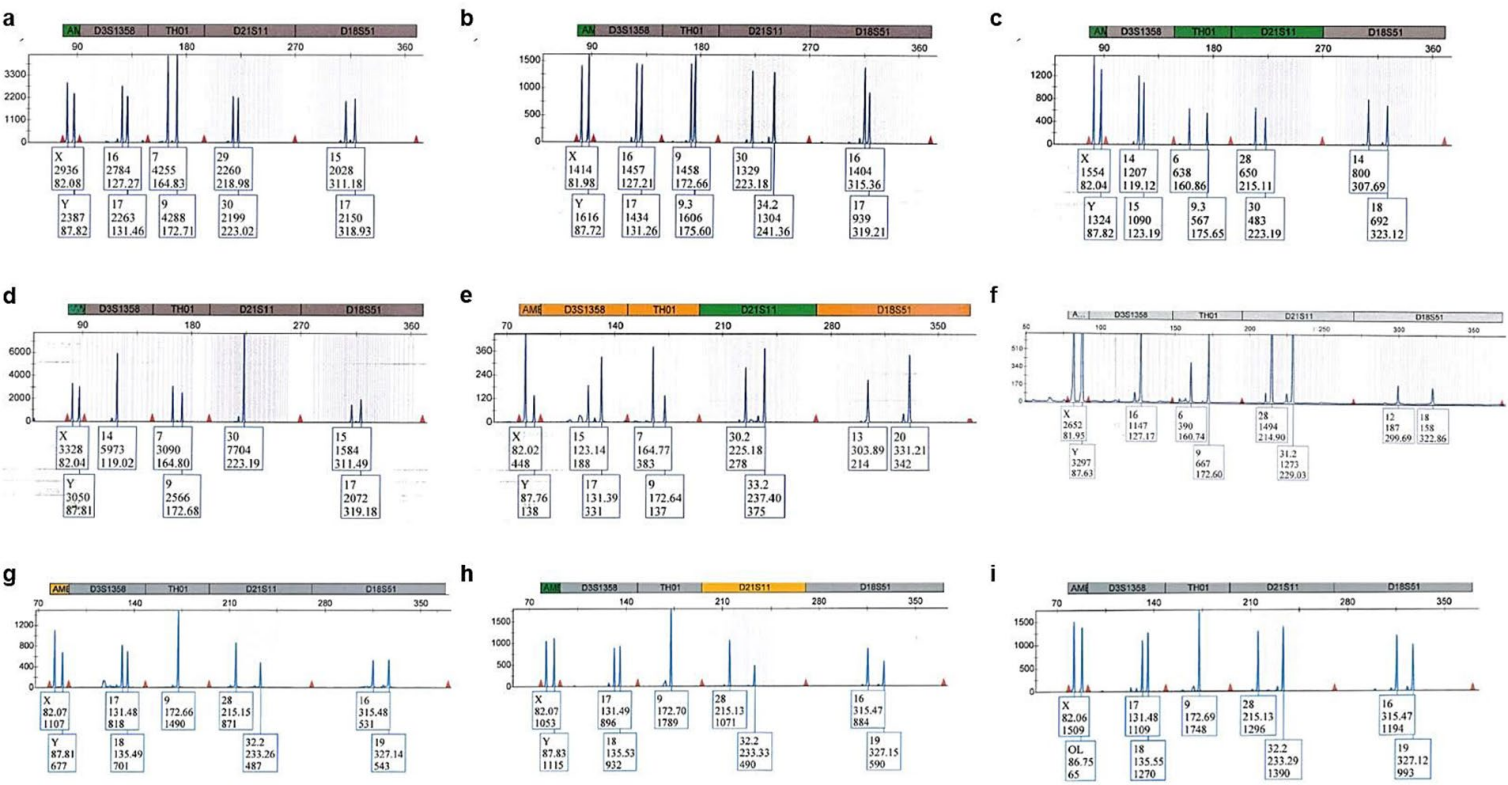
DNA profiles of charred bodies (blue dye channel, PowerPlex® ESX 17 System): **a** case 1, **b** case 2, **c** case 3, **d** case 4, **e** case 5, **f** case 6, or body fragments: **g** case 7, **h** case 8, and **i** case 9

The presence of inhibitors or artifacts was not observed in any sample. We obtained complete STR profiles for 23 autosomal loci (blue dye channel, PowerPlex® ESX 17 System; Fig. 7) and for 17 chromosome Y loci (data not shown). Therefore, the STR polymorphism results allowed us to successfully identify the cadavers based on the comparative material. The comparative analysis showed that spine fragments from cases 7–9 belonged to the same cadaver (Table 2).

**Table 2:** Statistical analysis of genetic testing done on DNA isolated from the intervertebral discs sampled from nine charred cadavers (Case 1–6) or charred body fragments (Case 7–9)

	**Case 1** **cadaver** **vs brother**	**Case 2** **cadaver vs parents**	**Case 3** **cadaver vs father**	**Case 4** **cadaver vs daughter**	**Case 5** **cadaver vs son**	**Case 6** **cadaver vs mother**	**Cases 7–9** **cadaver vs mother**
**Maternity Index**	-	3.578 × 10^7^	-	-	1.785 × 10^14^	4.611 × 10^7^	8.276 × 10^10^
**Probability of maternity (%)**	-	99.999997	-	-	99.9999999999994	99.999997	99.999999998
**Paternity Index**	-	1.622 × 10^15^	7.875 × 10^11^	2.412 × 10^7^	-	-	-
**Probability of paternity (%)**	-	99.9999999994	99.99999998	99.999995	-	-	-
**Likehood ratio**	6.787 × 10^8^	-	-	-	-	-	-
**Probability of siblings (%)**	99.9999998	-	-	-	-	-	-

The analysis of the results showed no relations between PMI and DNA concentration isolated from T. Large autosomal sequence (*p* = 0.493), T. Small autosomal sequence (*p* = 0.910), T. Y chromosome sequence (*p* = 0.574), and the DNA degradation index (*p* = 0.466). However, the statistically significant relationship was found between cadavers’ age and DNA concentration isolated from T. Large autosomal sequence (*r* =  −0.8224, *p* < 0.05), T. Small autosomal sequence (*r* =  −0.9040; *p* < 0.01), T. Y chromosome sequence (*r* =  −0.8738, *p* < 0.01), and the DNA degradation index (*r* =  −0.7891, *p* < 0.05).

## Discussion

The paper describes four cases where the victims died in a catastrophe: case 6, uncontrolled gas explosion; cases 7–9, uncontrolled nitroglycerin explosion. The recommendations of the International Society for Forensic Genetics (ISFG) specify the type of post-mortem samples that should be collected during post-mortem examination depending on the body condition. For severely burnt bodies, the ISFG recommends sampling: blood (on FTA card or swab) and buccal (mouth) swabs; blood and deep red muscle tissue if available (1.0 g), long compact bone (4–6-cm samples, using window cut without separating the shaft) and/or healthy teeth without fillings (molars preferable) and/or any available bone (10 g) if possible; dense cortical bone, or swab from inside the urinary bladder [18]. Research on exhumed bodies from World War II showed that it is possible to determine a complete genetic profile from the vertebral arch of the 12th thoracic vertebra [19]. Our research proves that it is possible to isolate good-quality DNA from discs derived from spine fragments and successfully identify victims. However, the presented paper does not intend to demonstrate that fibrocartilage of the intervertebral disc is more suitable than hard tissues but to show that it can be considered an alternative source material for faster and cheaper DNA isolation for personal identification.

Our research is not the first one describing the successful DNA isolation from the fibrocartilage of the intervertebral disc. Recently, Becker et al. demonstrated that possibility in 30 cases: 7 fresh cadavers, 10 in the early stage of decomposition, 6 in the advanced stage of decomposition, 3 exhumed bodies, 1 victim of drowning, 2 victims of severe burns, and 1 victim of the most severe burns. Since no other details were given, it can only be assumed that Becker et al. describe two fire victims with second- and third-degree burns and one fire victim with fourth-degree burns, respectively [11].

The temperature effect on DNA quality comprises quite a significant problem in forensic genetics. The quality of dental DNA is highly dependent on the temperature and duration of the heat exposure. All STR loci could be detected in teeth exposed to 100 °C for 60 min, but genetic identification was almost impossible from teeth exposed to 200 °C and above [20]. Maciejewska et al. [21] studied the possibility of obtaining DNA profiles from soft and hard tissues exposed to high temperatures for a short time. They were able to generate complete DNA profiles from soft tissues exposed to 900 °C for 5 min, but not from hard tissues. Literature data indicate that the average temperature in commercial cremation ovens ranges from 500 to 1250 °C, and no PCR products could be isolated after exposure to 600 °C and above [22–24].

In three out of 9 cases (cases 1–3) presented in this paper, an expert in the field of firefighting, appointed by the prosecutor conducting the investigation, assessed that the bodies could be exposed to temperatures ranging from > 660 to 1100 °C. However, no information on the duration of the heat exposure was provided by the expert. Since we were able to isolate DNA of substantial quantity and good quality and determine DNA profiles and confirm cadavers’ identity, we might assume that the exposure time was short. Unfortunately, the literature considerations on the high temperature exposure time in relation to DNA profile determination are selective and scarce [20].

The negative correlations between the DNA degradation index and the age of the cadavers indicate that the DNA quality decreases with age which negatively affects the possibility of DNA profiling in elderly victims. The above data seem to be consistent with the information about the disintegration of the mammalian disc genome increasing with age [25–27].

In the paper, we have presented the real influence of a thermal factor on the quality and quantity of DNA isolated from the intervertebral discs of cadavers. Using this source material, we proved that it is possible to isolate DNA and identify victims successfully. We also showed that the quality and quantity of isolated DNA decreases with victims’ age. However, we are aware that the presented paper’s main limitation is small number of cases and samples.

## Key Points

1. Human intervertebral disc fibrocartilage can serve as an alternative DNA source.
2. Cartilage tissue can be used for DNA profiling even in highly charred cadavers.
3. Charred torso fragments can also be used for genetic identification purposes.
4. DNA isolation from the intervertebral disc can be less time-consuming, labor-intensive, and more cost-effective compared to bone tissues.

## Data Availability

All data generated or analyzed during this study are available from the corresponding author on reasonable request.

## References

[1] Meng H, Zhang M, Xiao B (2019). Forensic age estimation based on the pigmentation in the costal cartilage from human mortal remains. Leg Med (Tokyo).

[2] Zhang K, Fan F, Tu M (2018). The role of multislice computed tomography of the costal cartilage in adult age estimation. Int J Legal Med.

[3] Pfeiffer H, Mörnstad H, Teivens A. Estimation of chronologic age using the aspartic acid racemization method. I. On human rib cartilage. Int J Legal Med. 1995;108(1):19–23. 10.1007/BF01845611. 10.1007/BF01845611 7495680

[4] Ohtani S, Matsushima Y, Kobayashi Y (2002). Age estimation by measuring the racemization of aspartic acid from total amino acid content of several types of bone and rib cartilage: a preliminary account. J Forensic Sci.

[5] Tomsia M, Nowicka J, Skowronek R (2020). A comparative study of ethanol concentration in costal cartilage in relation to blood and urine. Processes.

[6] Tomsia M, Nowicka J, Skowronek R, et al. Concentrations of volatile substances in costal cartilage in relation to blood and urine – preliminary studies. Arch Med Sadowej Kryminol. 2021;71. 10.5114/amsik.2021.106014. 10.5114/amsik.2021.106014 37376862

[7] Tomsia M, Głaz M, Nowicka J (2021). Sodium nitrite detection in costal cartilage and vitreous humor - case report of fatal poisoning with sodium nitrite. J Forensic Leg Med.

[8] Siriboonpiputtana T, Rinthachai T, Shotivaranon J (2018). Forensic genetic analysis of bone remain samples. Forensic Sci Int.

[9] Tomsia M, Droździok K, Javan G (2021). Costal cartilage ensures low degradation of DNA needed for genetic identification of human remains retrieved at different decomposition stages and different postmortem intervals. Postepy Hig Med Dosw.

[10] Droździok K, Tomsia M, Rygol K (2021). When DNA profiling is not enough? A case of same-sex siblings identification by odontological assessment after gas explosion-related building collapse. Leg Med (Tokyo).

[11] Becker J, Mahlke NS, Ritz-Timme S (2021). The human intervertebral disc as a source of DNA for molecular identification. Forensic Sci Med Pathol.

[12] Senst A, Scheurer E, Gerlach K (2021). Which tissue to take? A retrospective study of the identification success of altered human remains. J Forensic Leg Med.

[13] Grela M, Jakubczak A, Kowalczyk M, et al. Effectiveness of various methods of DNA isolation from bones and teeth of animals exposed to high temperature. J Forensic Leg Med. 2021;78:102131. 10.1016/j.jflm.2021.102131. 10.1016/j.jflm.2021.102131 33561692

[14] Schwark T, Heinrich A, Preusse-Prange A (2011). Reliable genetic identification of burnt human remains. Forensic Sci Int Genet.

[15] Chan JK (2014). The wonderful colors of the hematoxylin-eosin stain in diagnostic surgical pathology. Int J Surg Pathol.

[16] King DF, King LA (1986). A brief historical note on staining by hematoxylin and eosin. Am J Dermatopathol.

[17] Berent J. DNAStat, version 2.1, A computer program for processing genetic profile databases and biostatistical calculations. Arch Med Sadowej Kryminol. 2010;60(2–3):118–26. 21516944

[18] Prinz M, Carracedo A, Mayr WR (2007). DNA Commission of the International Society for Forensic Genetics (ISFG): recommendations regarding the role of forensic genetics for disaster victim identification (DVI). Forensic Sci Int Genet.

[19] Benedik Bevc T, Božič L, Podovšovnik E (2021). Intra-bone nuclear DNA variability and STR typing success in Second World War 12th thoracic vertebrae. Forensic Sci Int Genet.

[20] Lozano-Peral D, Rubio L, Santos I (2021). DNA degradation in human teeth exposed to thermal stress. Sci Rep.

[21] Maciejewska A, Włodarczyk R, Pawłowski R (2015). The influence of high temperature on the possibility of DNA typing in various human tissues. Folia Histochem Cytobiol.

[22] Absolonová K, Dobisiková M, Beran M (2012). The temperature of cremation and its effect on the microstructure of the human rib compact bone. Anthropol Anz.

[23] Bartelink EJ, Sholts SB, Milligan CF (2015). A case of contested cremains analyzed through metric and chemical comparison. J Forensic Sci.

[24] Zana M, Magli F, Mazzucchi A (2017). Effects of cremation on fetal bones. J Forensic Sci.

[25] Yousefzadeh M, Henpita C, Vyas R (2021). DNA damage-how and why we age?. E-life.

[26] Vo NV, Hartman RA, Patil PR (2016). Molecular mechanisms of biological aging in intervertebral discs. J Orthop Res.

[27] Mohanty S, Pinelli R, Pricop P (2019). Chondrocyte-like nested cells in the aged intervertebral disc are late-stage nucleus pulposus cells. Aging Cell.

